# Effects of the probiotic *Bifidobacterium animalis subsp*. *lactis* on the non-surgical treatment of periodontitis. A histomorphometric, microtomographic and immunohistochemical study in rats

**DOI:** 10.1371/journal.pone.0179946

**Published:** 2017-06-29

**Authors:** Milla S. T. Ricoldi, Flávia A. C. Furlaneto, Luiz F. F. Oliveira, Gustavo C. Teixeira, Jéssica P. Pischiotini, André L. G. Moreira, Edilson Ervolino, Maricê N. de Oliveira, Cristina S. B. Bogsan, Sérgio L. Salvador, Michel R. Messora

**Affiliations:** 1 Department of Oral and Maxillofacial Surgery and Periodontology, School of Dentistry of Ribeirao Preto, University of Sao Paulo–USP, Ribeirao Preto/ SP, Brazil; 2 Private Practice, Salvador/ BA, Brazil; 3 Department of Basic Sciences, Division of Histology, Dental School of Aracatuba, UNESP-Univ Estadual Paulista, Aracatuba/ SP, Brazil; 4 Department of Biochemical and Pharmaceutical Technology, School of Pharmaceutical Sciences, University of Sao Paulo–USP, Sao Paulo/ SP, Brazil; 5 Department of Clinical Analyses, School of Pharmaceutical Sciences of Ribeirao Preto, University of São Paulo—USP, Ribeirão Preto/ SP, Brazil; Virginia Commonwealth University, UNITED STATES

## Abstract

*Lactobacillus* probiotics have been investigated in periodontitis. However, the effects of the genus *Bifidobacterium* on periodontitis are hardly known. This study evaluated the effects of the probiotic (PROB) *Bifidobacterium animalis subsp*. *lactis* (*B*. *lactis*) HN019 as an adjunct to scaling and root planing (SRP) in rats with experimental periodontitis (EP). At baseline, 32 rats were assigned to 4 groups: C (control), PROB, EP-SRP and EP-SRP-PROB. In groups EP-SRP and EP-SRP-PROB, the mandibular first molars of the animals received a ligature. At day 14, the ligatures were removed and SRP was performed. Animals of groups PROB and EP-SRP-PROB were orally administered with 10 mL/day of 10^9^ colony forming units of *B*. *lactis* HN019 for 15 days, starting at day 14. Animals were euthanized at day 29. Histomorphometric, microtomographic and immunohistochemical analyses were performed. Microbiological effects of *B*. *lactis* on biofilm were also evaluated. Data were statistically analyzed (ANOVA, Tukey; Kruskal-Wallis, Dunn’s; Two-tailed t-test; *p*<0.05). Group EP-SRP-PROB presented reduced alveolar bone resorption and attachment loss when compared with Group EP-SRP (*p*<0.05). Group EP-SRP-PROB showed significantly fewer osteoclasts, increased expression of anti-inflammatory cytokines and reduced expression of proinflammatory cytokines compared with Group EP-SRP (*p*<0.05). *B*. *lactis* promoted a higher ratio between aerobic and anaerobic bacteria in biofilm samples (*p*<0.05). *B*. *lactis* HN019 may have a role in the treatment of EP in rats, as an adjunct to SRP.

## Introduction

There is a need for the development of alternative and/or adjunctive therapies to the conventional periodontal treatment [1]. The use of beneficial bacteria has arisen as a potential concept in the prevention and treatment of periodontal diseases [2]. According to the World Health Organization, probiotics are live microorganisms that can confer health benefits to the host when consumed in adequate amounts [3]. The mechanisms of action of probiotics are not completely understood but they seem to modulate host immunoinflammatory response and influence the structure and function of microbiota [1,4].

The studies that evaluated the effects of probiotics on the treatment of periodontal diseases demonstrated that they can reduce periodontopathogens, improve periodontal clinical parameters, decrease the levels of proinflammatory cytokines and potentiate the effects of scaling and root planing (SRP) [2,5–8].

Although the scientific literature presents promising results, it is meaningful to consider that the results obtained with probiotics can not be extrapolated [9] since they depend on the strain, dose, frequency and route of administration used. The researches which evaluated the effects of probiotics on periodontal diseases to date used primarily bacteria from the genus *Lactobacillus*. However, other potential probiotics deserve investigations. *Bifidobacterium animalis subsp*. *lactis* (*B*. *lactis*) is part of the human microbiota and presents a symbiotic relationship with the host. It is considered a potential probiotic because it possesses immunomodulatory [10–12] and antimicrobial [13–15] properties.

High quality preclinical studies, such as the use of animal models of periodontitis, may provide important data about the safety and efficacy of probiotics [16]. Recently, our group evaluated the topical use of one strain of *B*. *lactis*, named HN019, on the development of experimental periodontitis (EP) in rats. It was observed that the treatment promoted a protective effect against periodontal breakdown, modifying immunoinflammatory and microbiological parameters [17]. To the best of our knowledge, there are no studies evaluating the effects of *B*. *lactis* HN019 together with SRP on periodontitis. Therefore, the purpose of this study was to evaluate the histomorphometric, microtomographic and immunohistochemical outcomes following the administration of *B*. *lactis* HN019 as an adjunct to SRP in rats with ligature-induced periodontitis. Microbiological effects of *B*. *lactis* HN019 on biofilm during the development of EP were also evaluated.

## Materials and methods

### Sample

This study was performed after review and approval by the Ethics Committee on Animal Experimentation at School of Dentistry of Ribeirao Preto, University of Sao Paulo–FORP/USP (protocol 13.1.136.53.5). Authors followed the ARRIVE (Animal Research: Reporting of In Vivo Experiments) guidelines.

The sample size was determined to provide 80% power to recognize a significant difference of 20% among groups and the standard deviation of 15% with a 95% confidence interval (α = 0.05), considering the change in the alveolar bone in the furcation area (Area of No Bone—ANB) as the primary outcome variable. Thus, a sample size of eight animals per group was needed.

### Experimental model

Thirty-two adult male Wistar rats (*Rattus norvegicus*, *albinus*), weighing 230–250 g, were used (Central Animal Facility, FORP/USP). The rats were kept in a 12-hour light/dark cycle and temperatures between 22 and 24°C. They were housed in individual metabolic cages and fed with selected solid diet and water *ad libitum*. Rats were assigned to the following groups (n = 8): Group C (control); Group PROB (probiotic); Group EP-SRP (experimental periodontitis + scaling and root planing) and Group EP-SRP-PROB (experimental periodontitis + scaling and root planing + probiotic). All analyses were performed by calibrated and blinded examiners.

### Induction of EP

In Groups EP-SRP and EP-SRP-PROB, rats were anesthetized by an intraperitoneal injection of xylazine (Rompum^®^, Bayer Animal Health, Sao Paulo, SP, Brazil; 10 mg/kg body weight) and ketamine (Dopalen^®^, Agribands Purina do Brasil Ltda., Paulinia, SP, Brazil; 80 mg/kg body weight). A cotton ligature was placed around their right mandibular first molars, as previously described [18].

### SRP procedures

After 14 days, the animals were anesthetized, as previously described, and the ligatures (groups EP-SRP and EP-SRP-PROB) were removed. The right mandibular first molars of the animals of these groups were subjected to SRP with Mini Five 1–2 curettes (Hu-Friedy Co. Inc., Chicago, IL, USA) through ten distal-mesial traction movements on both buccal and lingual surfaces. The furcation and interproximal areas were scaled with the same curettes through cervico-occlusal traction movements [19].

### Probiotic therapy

Starting from day 14 of the experiment, 10% skimmed milk (Molico^®^, Nestle Brasil Ltda., Sao Paulo, SP, Brazil) was orally administered to the animals once a day (at 8:00 am) during 15 days. For Groups PROB and EP-SRP-PROB, 10% skimmed milk were inoculated with *B*. *lactis* HN019 (Howaru Bifidus Lyo 40 DCU, DuPont™ Danisco^®^/Fermentech, Sao Paulo, SP, Brazil) culture to obtain 10^9^ colony-forming units (CFU)/10 mL. In Group EP-SRP-PROB, probiotic therapy was initiated immediately after SRP. The animals of Groups C and EP-SRP received 10 mL of 10% skimmed milk with placebo (without *B*. *lactis* HN019).

### Euthanasia

Animals were euthanized with a lethal dose (150 mg/kg body weight) of sodium thiopental (Thiopentax^®^, Cristalia Produtos Quimicos Farmaceuticos Ltda., Sao Paulo, SP, Brazil) at day 29 of the experiment. The hemimandibles were excised and tissue samples from distinctive parts of the small intestines (duodenum, jejunum, ileum) were collected. All specimens were fixed in 4% formaldehyde for 24 hours.

### Microcomputed tomography (micro-CT) analyses

Non-demineralized specimens were scanned by a cone-beam micro-CT system (Skyscan 1172, Bruker, Kontich, Belgium). The x-ray generator was operated at an accelerated potential of 60 kV with a beam current of 165 μA and an exposure time of 650 ms per projection. Images were produced with a voxel size of 6x6x6 μm.

Using an appropriated software (Data Viewer^®^, version 1.5.0, Bruker, Kontich, Belgium), the generated 3 dimensional models were rotated into a standard position as the following criteria: (1) in transaxial plane, the mandibular first molar (M1) had its axis vertically positioned, (2) in coronal plane, the mandibular bone was vertically orientated, with the mesial root of the M1 in the upper position of the image and (3) in sagittal plane, the occlusal surface of M1 was horizontally positioned [19]. Linear measurements on alveolar bone level (ABL) were performed at four different sites: buccal, lingual, interproximal and furcation [19]. For buccal, lingual and interproximal sites, the linear distances from cementoenamel junction (CEJ) to alveolar bone crest (ABC) were measured [20]. For the interproximal site, coronal dataset was analyzed using appropriated software (CT-Analyser^®^, version 1.13.5.1+, Bruker, Kontich, Belgium) [19]. For the furcation site, ABL was assessed by measuring the distance between the roof of the furcation and the ABC in the furcation area. The four linear measurements obtained from each animal were summed to express the ABL value [19].

For volumetric measurements, a volume of interest (VOI-prismatic section) was outlined from the apexes of all roots of M1 up to the roof of the furcation of M1, touching the roots surfaces, in all images of the coronal dataset, using the software CT-Analyser^®^ [19]. The following parameters were analyzed: i) Bone Volume (BV): percentual of the VOI filled with bone tissue; ii) Mean Bone Porosity (BP): percentual of bone porosity present in the VOI [19].

### Histomorphometric analysis of periodontal tissues

After routine laboratorial processing [18], two sections representing the most central buccal-lingual portion in the furcation area of right mandibular first molars were selected for histopathological and histometric analyses. The histopathological analysis was performed by a certified histologist (E.E.) using a light microscope.

For histometric analysis, photomicrographs were captured by a digital camera (DM2000, Leica Microsystems, Wetzlar, Germany) connected to a light microscope (DFC295, Leica Microsystems, Wetzlar, Germany). The images were analyzed using appropriate software (Diracon Bio Informatica Ltda., Vargem Grande do Sul, SP, Brazil). The ANB and the attachment loss (AL) were measured [18,20].

### Immunohistochemical analyses of periodontal tissues

Immunohistochemical processing was performed through the indirect immunoperoxidase method, as described elsewhere [21]. The histological slides containing samples from all animals were incubated with one of the following primary antibodies: rabbit anti-interleukin (IL)-1β (1:100-Rabbit anti-IL-1β–SC 7884, Santa Cruz Biotechnology, Santa Cruz, CA, USA), goat anti-cytokine-induced neutrophil chemoattractant–CINC (1:100-Goat anti-CINC1–ab 10365, abcam^®^, Cambridge, MA, USA), goat anti-IL-10 (1:100-Goat anti-IL-10–SC 1783, Santa Cruz Biotechnology), rabbit anti-transforming growth factor (TGF)-β1 (1:200-Rabbit anti-TGF-β1–SC 146, Santa Cruz Biotechnology) or goat anti-tartrate-resistant acid phosphatase–TRAP (1:100-Goat anti-TRAP–SC 30833, Santa Cruz Biotechnology). Histologic sections were evaluated under light microscopy with an optical microscope (Axiovision 4.8.2, Carl Zeiss MicroImaging GmbH, Jena, Germany).

A quantitative immunolabeling analysis of TRAP-positive cells and semi-quantitative immunolabeling analyses of IL-1β, CINC, IL-10 and TGF-β1 were performed in the entire furcation region at 400x magnification [20]. For semi-quantitative analyses, the following scores were determined: score 0–no immunolabeling (total absence of immunoreactivity in the area); score 1–low immunolabeling pattern (≈ ⅓ of the area presenting immunoreactivity); score 2–moderate immunolabeling pattern (≈ ⅔ of the area presenting immunoreactivity) and score 3–high immunolabeling pattern (almost the totality of the area presenting immunoreactivity) [19].

### Histomorphometric analysis of small intestine

The collected samples were routinely processed [18]. For histometric analyses, the measurements included villous height (VH) and crypt depth (CD), as previously described [18].

### Effects of B. lactis HN019 on ligature-associated microbiota

Twelve adult male Wistar rats (*Rattus norvegicus*, *albinus*), weighing 230-250g, not used in the experiments previously described, were used. Animals were divided into groups Placebo (n = 6) and Intervention (n = 6). A cotton ligature was placed around their right mandibular first molars, as described by Messora et al. (2013) [18]. 10% skimmed milk (Molico^®^) was orally administered to the animals once a day during 15 days. In Intervention Group, the milk was inoculated with *B*. *lactis* HN019 (Howaru Bifidus Lyo 40 DCU) culture to obtain 10^9^ CFU/10 mL.

The ligatures were recovered from the rats at day 15 and incubated under aerobic and anaerobic conditions for 7 days at 35°C for enumeration of total cultivable CFU [1]. The ratio between aerobic and anaerobic bacteria was calculated in each group.

### Statistical analyses

Normality and homoscedasticity of the data were verified. The significance of differences among groups in relation to the immunolabeling pattern of IL-1β, CINC, IL-10 and TGF-β1 was determined by Kruskal-Wallis tests, followed by Dunn’s multiple comparison post-test. The data from the microtomographic and histometric analyses were assessed by analysis of variance (ANOVA) followed by *post-hoc* Tukey test. Two-tailed t-test was performed to evaluate the microbiologic data. The significance level was set at 5% in all tests.

## Results

### Micro-CT analyses

**Fig 1:**
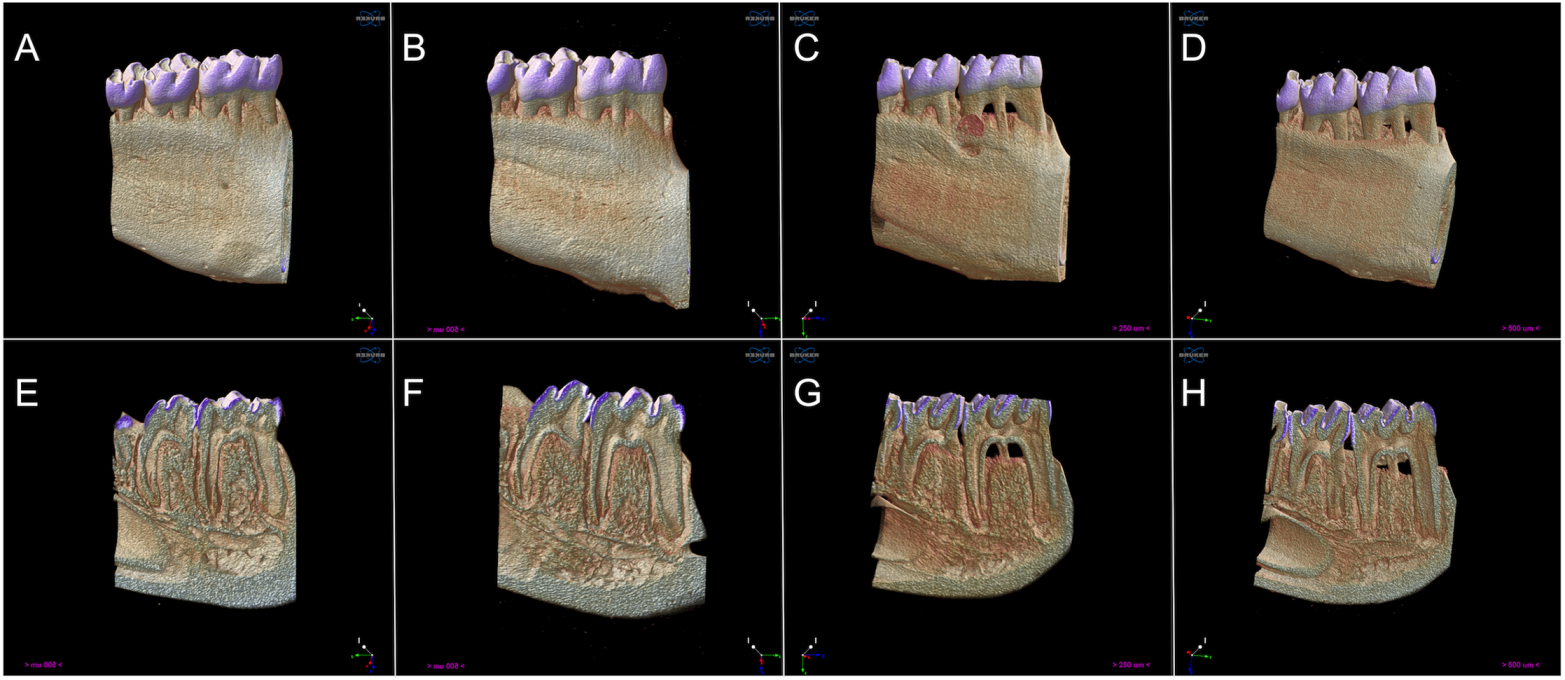
Microtomographic analysis. Three-dimensional rendered reconstructions of the microtomographic sections of groups C (A, E), PROB (B, F), EP-SRP (C, G) and EP-SRP-PROB (D, H). Buccal view (A-D). Internal surface view, sagittal section (E-H). Pixel size = 7.96 μ.

Group EP-SRP-PROB presented greater BV and reduced BP and ABL when compared with Group EP-SRP (ANOVA, *p*<0.05; Fig 1). Means and standard deviations of ABL, BV and BP, as well as intergroup comparisons, are depicted in Table 1.

**Table 1:** Micro-CT analyses. Means and standard deviations (SD) of BV (%), BP (%) and ABL (μm) of Groups C, PROB, EP-SRP and EP-SRP-PROB, with comparisons among groups.

VARIABLE	EXPERIMENTAL GROUPS			
	C N = 8	PROB N = 8	EP-SRP N = 8	EP-SRP-PROB N = 8
	Mean ± SD	Mean ± SD	Mean ± SD	Mean ± SD
**BV (%)**	80.3 ± 9.3^*^	83.9 ± 2.5^*^	31.8 ± 14.1	67.1 ± 20.9^*^
**BP (%)**	19.3 ± 9.3^*^	17.1 ± 2.9^*^	70.5 ± 16.9	22.8 ± 17.8^*^
**ABL (**μ**m)**	1537 ± 331.8^*^	1754 ± 610.9^*^	4682 ± 1634	2853 ± 820.6^*^

*Significant difference when compared with Group EP-SRP (*p*<0.05)

BV = bone volume; BP = bone porosity; ABL = alveolar bone level.

### Histomorphometric analysis of periodontal tissues

**Fig 2:**
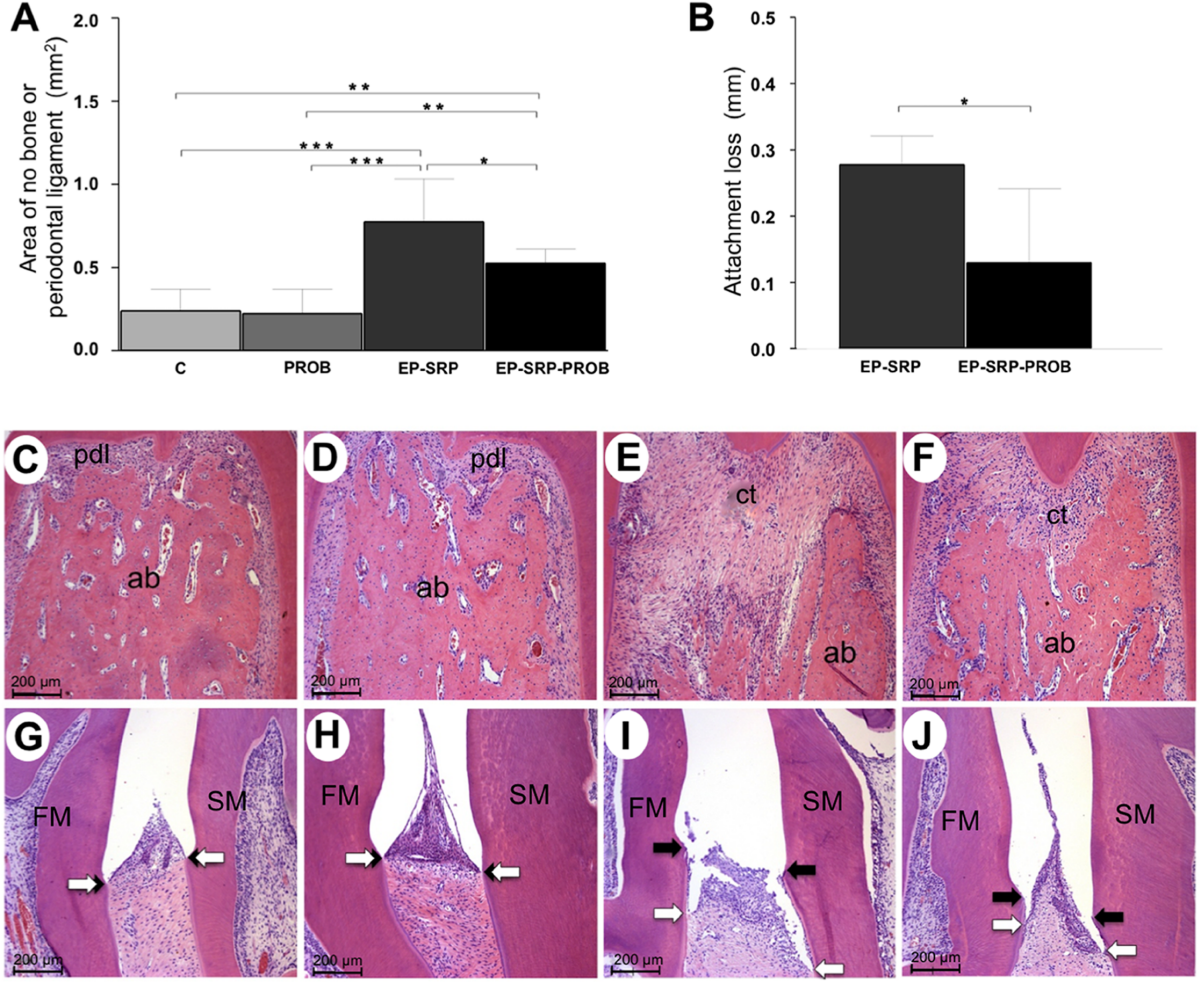
Histomorphometric analysis of periodontal tissues. Means and standard deviations of ANB (A; furcation area) and AL (B; interproximal area), with comparisons among groups. Photomicrographs of periodontal tissues in the furcation (C-F) and interproximal areas (G-J) of mandibular first molars: Group C (C and G); Group PROB (D and H); Group EP-SRP (E and I); Group EP-SRP-PROB (F and J). Abbreviations and symbols: ab = alveolar bone; ct = connective tissue; pdl = periodontal ligament; ANB = area of no bone; AL = attachment loss; FM = first molar; SM = second molar; black arrows = cementoenamel junction; white arrows = epithelial attachment; * = *p*<0.05; ** = *p*<0.01; *** = *p*<0.001. Scale bars: C-J = 200 μm. (Hematoxylin and Eosin stain).

Groups with EP showed greater alveolar bone resorption and AL than Groups C and PROB (*p*<0.05; Fig 2A and 2B), which did not present AL. When compared with Group EP-SRP, Group EP-SRP-PROB presented ANB and AL significantly reduced (*p*<0.05; Fig 2A and 2B).

In Groups C and PROB, periodontal ligament (PL) presented a great amount of collagen fibers, fibroblasts and blood vessels. Collagen fibers were embedded both in cementum and in alveolar bone. There were a few irregularities on bone tissue of the furcation area (Fig 2C and 2D). In both groups, junctional and sulcular epithelia integrities were observed in the interproximal area (Fig 2G and 2H).

In Group EP-SRP, connective tissue presented moderate inflammatory infiltrate consisting primarily of neutrophils in the furcation region. The bone of the interradicular septum showed irregular outer contours (Fig 2E) due to the presence of numerous active osteoclasts. An intense destruction of the junctional and sulcular epithelia was observed in the interproximal area (Fig 2I).

In Group EP-SRP-PROB, the inflammatory infiltrate in the furcation region was mainly composed of neutrophils and was more restricted than the one observed in Group EP-SRP. Bone tissue in the interradicular septum presented irregular external contour and was covered with osteoblasts or bone lining cells. Few active osteoclasts were observed. Bone neoformation within the interradicular septum was observed in the majority of the specimens (Fig 2F). The junctional and sulcular epithelia of the interproximal area were less injured than that of Group EP-SRP (Fig 2J).

### Immunohistochemical analyses of periodontal tissues

**Fig 3:**
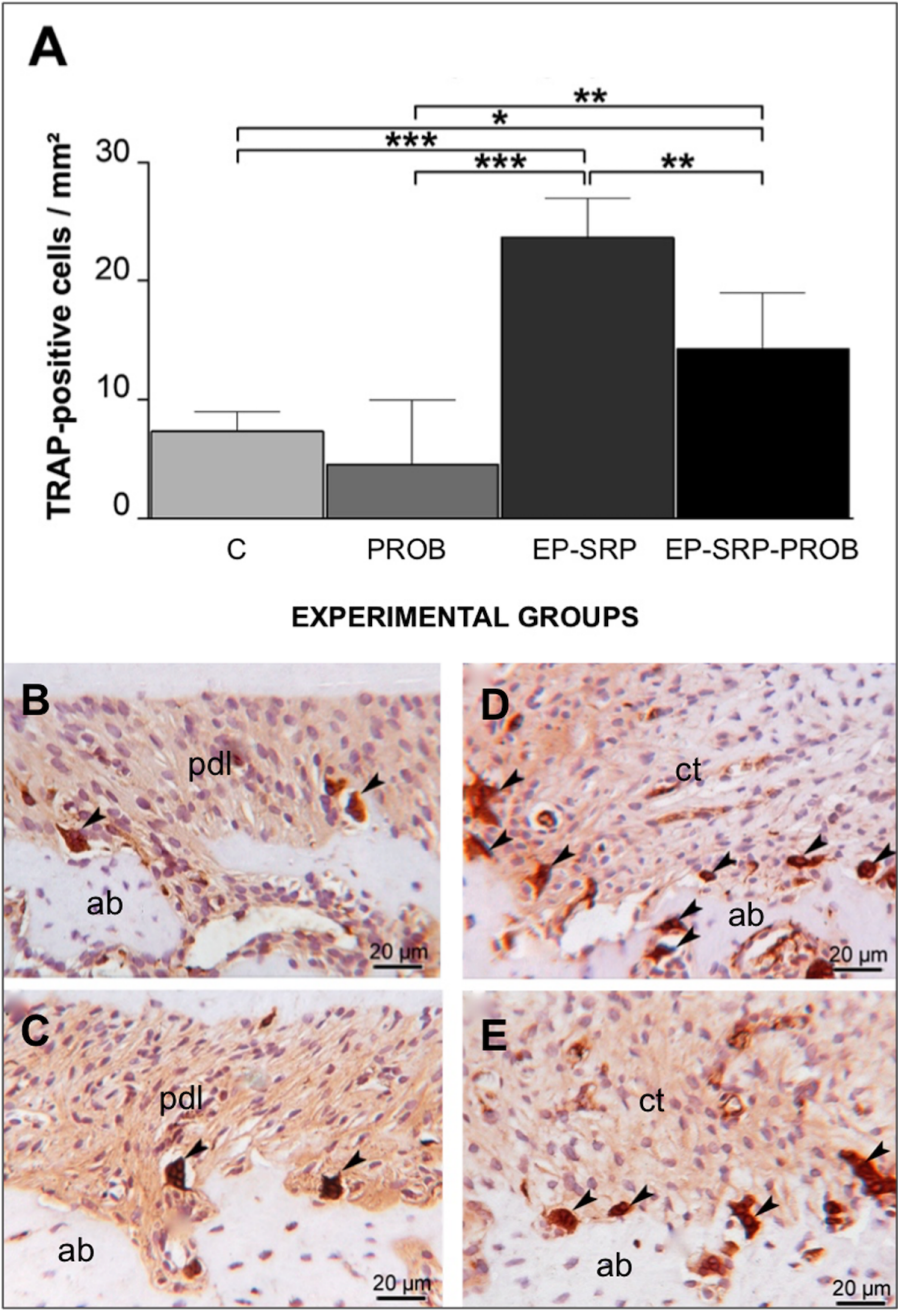
Immunohistochemical analyses–TRAP. Means and standard deviations of the number of TRAP-positive multinucleated cells (A), with comparisons among groups. Photomicrographs demonstrating immunolabeling for TRAP (B-E) in the furcation regions of mandibular first molars: Group C (B); Group PROB (C); Group EP-SRP (D); Group EP-SRP-PROB (E). Abbreviations and symbols: ab = alveolar bone; ct = connective tissue; pdl = periodontal ligament; black arrowhead = TRAP-positive multinucleated cell; * = *p*<0.05; ** = *p*<0.01; *** = *p*<0.001. Scale bars: B-E = 20 μm. (Hematoxylin counterstaining).

The number of TRAP-positive multinucleated cells was greater in Groups EP when compared with Groups C and PROB (*p*<0.05; Fig 3). Group EP-SRP-PROB presented less TRAP-positive multinucleated osteoclasts than Group EP-SRP (*p*<0.05; Fig 3).

**Fig 4:**
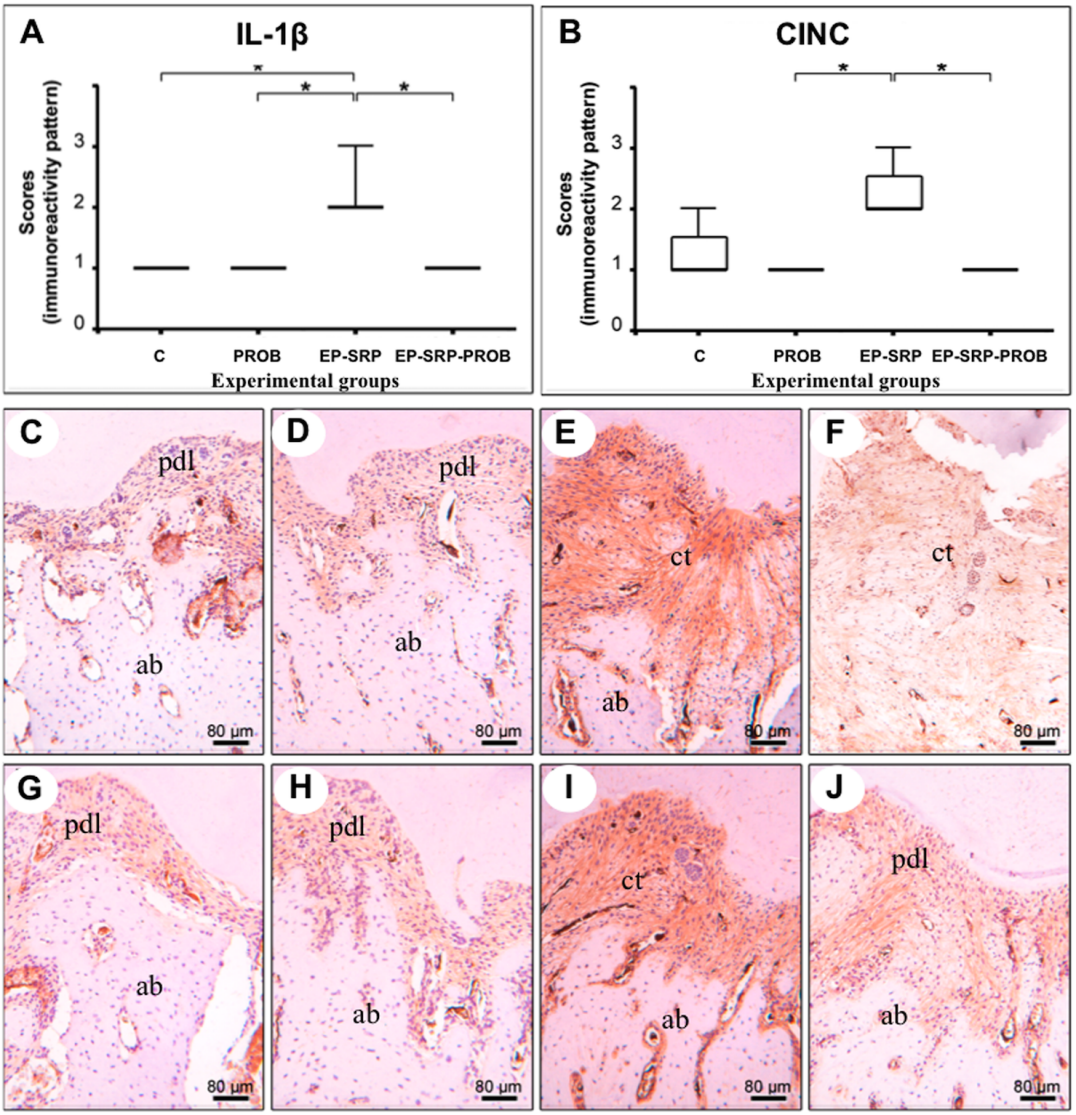
Immunohistochemical analyses—IL-1β and CINC. Medians, interquartile range and maximum and minimum values of the immunolabeling scores for IL-1β (A) and CINC (B), with comparisons among groups. Photomicrographs showing immunolabeling for IL-1β (C-F) and CINC (G-J) in the furcation regions of mandibular first molars: Group C (C and G); Group PROB (D and H); Group EP-SRP (E and I); Group EP-SRP-PROB (F and J). Abbreviations: ab = alveolar bone; ct = connective tissue; pdl = periodontal ligament; * = *p*<0.05. Scale bars: C-J = 80 μm. (Hematoxylin counterstaining).

**Fig 5:**
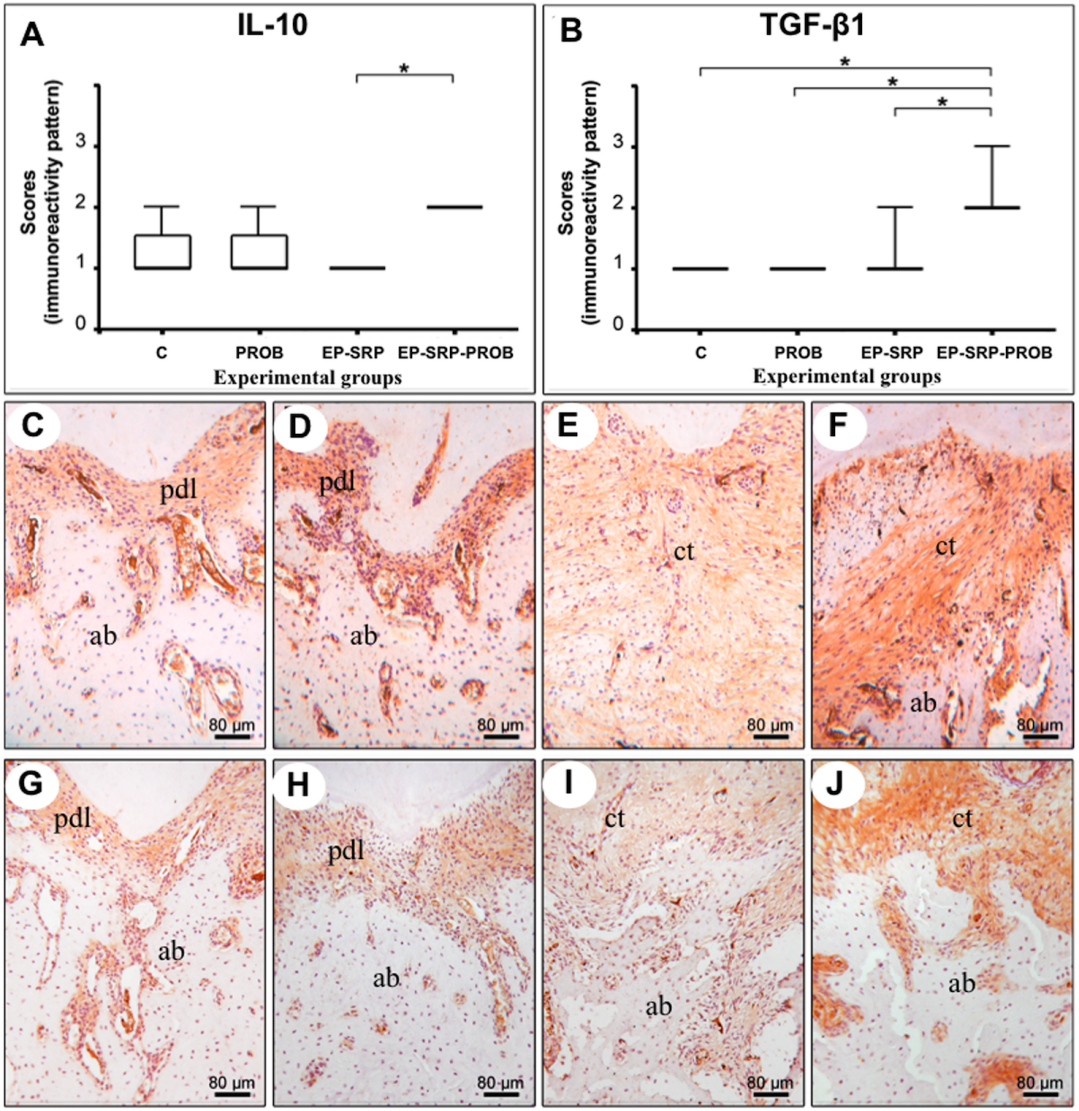
Immunohistochemical analyses—IL-10 and TGF-β1. Medians, interquartile range and maximum and minimum values of the immunolabeling scores for IL-10 (A) and TGF-β1 (B), with comparisons among groups. Photomicrographs showing immunolabeling for IL-10 (C-F) and TGF-β1 (G-J) in the furcation regions of mandibular first molars: Group C (C and G); Group PROB (D and H); Group EP-SRP (E and I); Group EP-SRP-PROB (F and J). Abbreviations: ab = alveolar bone; ct = connective tissue; pdl = periodontal ligament; * = *p*<0.05. Scale bars: C-J = 80 μm. (Hematoxylin counterstaining).

Group EP-SRP-PROB presented lower immunolabeling patterns for IL-1β and CINC (Fig 4) and higher immunolabeling patterns for TGF- β1 and IL-10 (Fig 5) than Group EP-SRP (*p*<0.05).

### Histomorphometric analysis of small intestine

**Fig 6:**
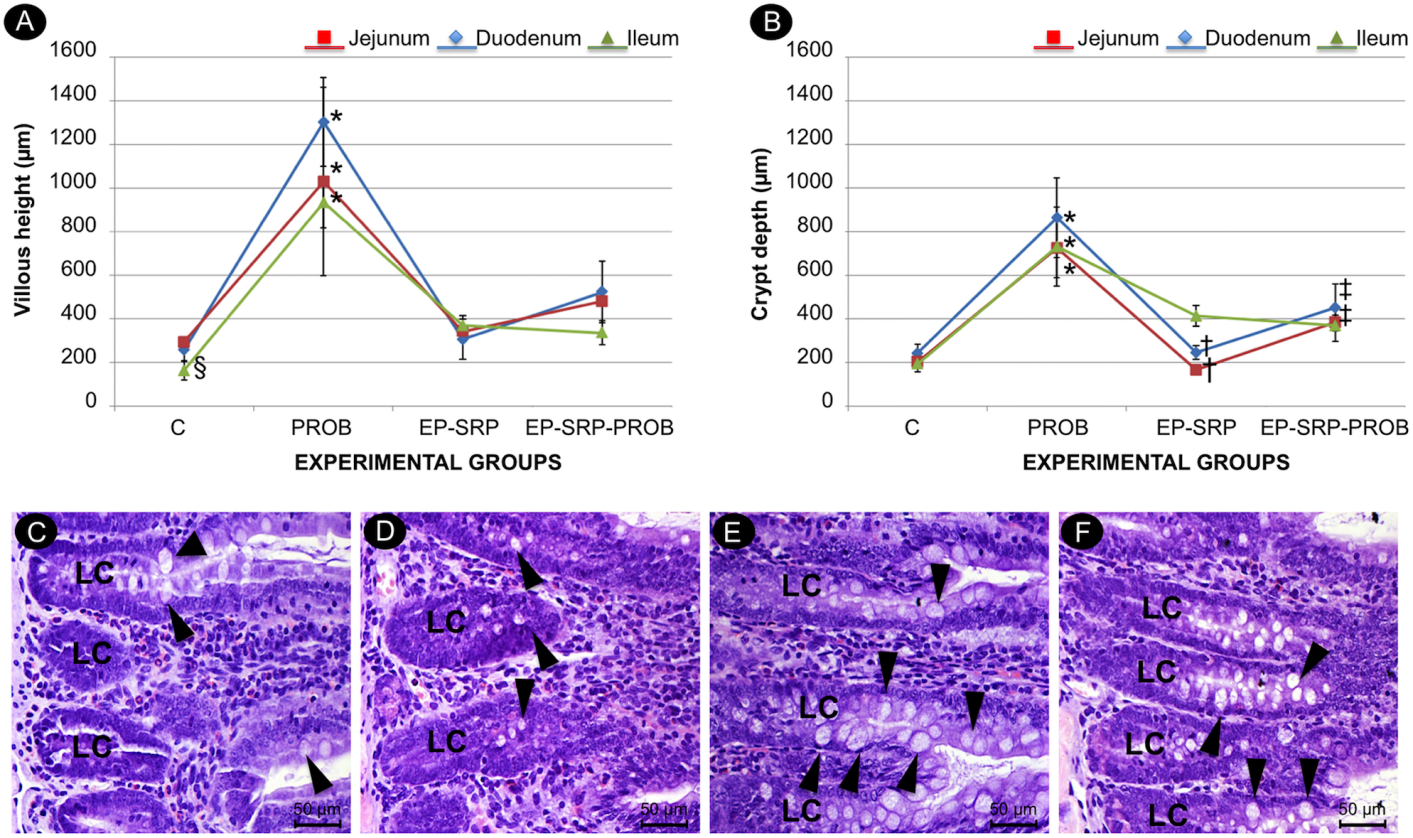
Histomorphometric analysis of small intestine. Mean values and standard deviations of VH (A) and CD (B) in intestinal sections, with comparisons among groups. Photomicrographs of small intestine (duodenum sections): Group C (C); Group EP-SRP (D); Group PROB (E); Group EP-SRP-PROB (F). Abbreviations and symbols: LC = crypt of Lieberkühn; VH = villous height; CD = crypt depth; black arrowhead = calciform cells; * = Significant difference (*p*<0.05) when compared with Groups C, EP-SRP and EP-SRP-PROB; ^†^ = Significant difference (*p*<0.05) between Groups EP-SRP and EP-SRP-PROB; ^‡^ = Significant difference (*p*<0.05) between Groups C and EP-SRP-PROB; ^§^ = Significant difference (*p*<0.05) between Groups C and EP-SRP. Scale bars: C-F = 50 μm. (Hematoxylin and Eosin stain).

Group PROB presented greater VH and CD in duodenum, jejunum and ileum samples than the other groups (*p*<0.05, Fig 6A and 6B). Group EP-SRP demonstrated VH greater than Group C in ileum samples (*p*<0.05, Fig 6A). Group EP-SRP-PROB presented greater CD in duodenum and jejunum samples than Group EP-SRP (*p*<0.05, Fig 6B).

A normal intestinal structure was observed in all groups, characterized by a mucosal layer presenting villosities covered with simple cylindrical epithelium, calciform cells and crypt of Lieberkühn. In groups PROB, the crypt of Lieberkühn and villosities were developed and presented a greater number of calciform cells when compared with the other groups in duodenum (Fig 6C–6F), jejunum and ileum samples.

### Effects of B. lactis HN019 on ligature-associated microbiota

**Fig 7:**
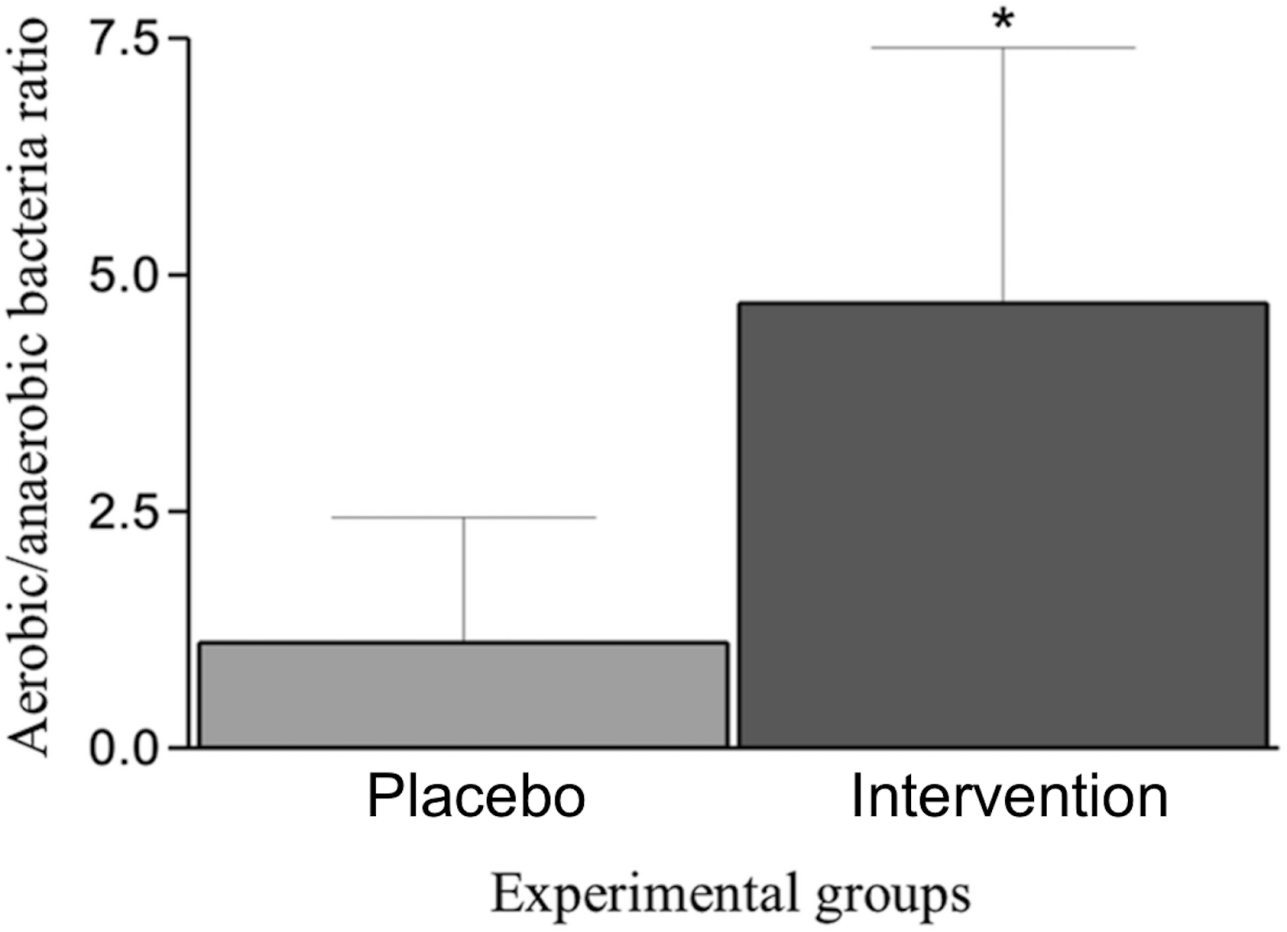
Effects of *B*. *lactis* HN019 on ligature-associated microbiota. Means and standard deviations of the ratios between aerobic and anaerobic bacteria in groups Intervention and Placebo. * = Significant difference (*p*<0.05) when compared with Group Placebo.

The ratio between aerobic and anaerobic bacteria was higher in Intervention Group when compared with Placebo Group (*p*<0.05). Means and standard deviations of the ratio are reported in Fig 7.

## Discussion

The use of natural and non-invasive approaches to prevent and treat oral diseases is attractive, and could also avoid concerns regarding the use of pharmacological therapies, such as antibiotics [22]. In this study, the probiotic therapy potentiated the effects of SRP in periodontitis treatment in rats. It influenced the balance of proinflammatory (IL-1β and CINC) and anti-inflammatory (IL-10 and TGF-β1) cytokines and reduced the number of osteoclasts, attachment and alveolar bone losses, the local inflammation as well as favored the tissue repair.

To the authors’ knowledge, this is the first study to administrate the genus *Bifidobacterium* as an adjunct to SRP in periodontitis. The use of this selected strain was supported by an in vitro study [23]. These authors showed that bifidobacteria could survive in saliva and bind to *Fusobacterium nucleatum*-covered hydroxyapatite. This finding may be a critical step for a successful periodontal therapy, since this co-aggregation may decrease the number of periodontopathogens in oral biofilm. In fact, the most important function of *Fusobacterium nucleatum* in subgingival bacterial biomass appears to be its capacity to facilitate the co-aggregation of other periodontal pathogens, such as *Aggregatibacter actinomycetemcomitans*, *Porphyromonas gingivalis* and *Tannerella forsythia* [24]. In the present study, the subgingival biofilm of the animals non-ligated (Groups C and PROB) or treated with SRP (Groups EP-SRP and EP-SRP-PROB) were not evaluated due to the inherent limitations of the experimental model. Without the presence of ligatures in Wistar rats, it is difficult to collect an adequate amount of oral biofilm for appropriate microbiologic analyses. Therefore, a parallel experiment was conducted to evaluate the effects of *B*. *lactis* HN019 on the microbiota associated with the ligature during the development of EP in rats. It was observed that this probiotic agent modulated the oral microbiota in a way that favored the growth of aerobic bacteria at the expense of anaerobic bacteria. As periodontitis-associated bacteria are predominantly anaerobic [25], this finding suggests that *B*. *lactis* HN019 treatment may inhibit periodontitis, at least in part, through modulation of the periodontal microbiota. In fact, the animals treated with *B*. *lactis* HN019 in this parallel experiment presented less alveolar bone resorption than the animals not treated, in morphometric analysis (data shown in S1 Fig).

In this study, a reduced expression of IL-1β and an increased expression of IL-10 were observed in Group EP-SRP-PROB when compared with Group EP-SRP. Higher levels of IL-10 were also observed in colon of gnotobiotic mice challenged with *Salmonella typhimurium* and treated with *B*. *lactis* [26]. IL-10 is capable of inhibiting IL-1β and tumor necrosis factor-α, which present synergistic actions on inflammatory processes, amplifying the host response [27]. The increased ratio IL-1β/IL-10 in gingival crevicular fluid can be associated with the progression of aggressive periodontitis [28].

The probiotic therapy as an adjunct to SRP also reduced the magnitude of the inflammatory response and improved tissue repair in Group EP-SRP-PROB, increasing the expression of TGF-β1 in periodontal tissues. Previous studies have already demonstrated that *B*. *lactis* HN019 can increase TGF-β1 levels in breast milk [29] and in blood [30]. TGF-β suppresses collagenase production by fibroblasts and macrophages, enhances the expression of tissue inhibitors of matrix-metalloproteinases (MMPs), increases the synthesis of extracellular matrix molecules and promotes chemoattractant effects on bone cells [31].

A reduced number of TRAP-positive multinucleated cells was found in Group EP-SRP-PROB when compared with Group EP-SRP. This may be explicated in part by the reduced expression of CINC (homolog of the human IL-8) in periodontal tissues of the animals treated with probiotic. Liu et al. (2010) [32] demonstrated that *B*. *lactis* HN019 exerts anti-inflammatory effects on the epithelium by down-regulating the secretion of IL-8. IL-8 has been implicated in a wide variety of diseases and is an important proinflammatory cytokine and immunomodulatory factor, which can induce osteoclast differentiation/maturation and the maintenance of bone resorption activity involved in periodontal tissue destruction [33].

Group PROB presented greater CD and VH in duodenum, jejunum and ileum samples when compared with the other groups. Also, Group EP-SRP-PROB showed greater CD than Group EP-SRP in duodenum and jejunum samples. In fact, probiotics can improve the host mucosal barrier function leading to diminished immunologic reactivity, displacing deleterious microbes from the mucosal surface and modulating the mucosal immune system [34]. In a previous study of this group [18], it was demonstrated that probiotic supplementation protected the small intestine from reactive changes induced by EP. It is important to punctuate that a diminished intestinal inflammation might be related to an improved periodontal health. Grossner-Schreiber et al. (2006) [35] reported an increased prevalence of periodontal disease in patients with inflammatory bowel disease.

The present study provided a proof-of-concept that the probiotic *B*. *lactis* HN019 presents therapeutic potential in periodontitis treatment. Nevertheless, it was not possible to determine whether the effects of this probiotic strain on periodontitis occurred mainly because of antimicrobial or immunomodulatory properties. The findings of the present study need to be confirmed with more advanced experimental models in the phylogenetic scale and in clinical trials. It is also mandatory to analyze the influence of the vehicle by which this probiotic strain is administered in its therapeutic potential and oral colonization as well as its survival in the oral cavity. Besides, more studies are required to evaluate other modes of application and different therapeutic regimens.

## Conclusion

It can be concluded that the oral administration of *B*. *lactis* HN019 potentiates the effects of SRP in the treatment of EP in rats.

## Supporting information

S1 Fig Morphometric analysis. (A) Means and standard deviations of the Area (mm2) of No Bone (delimited in the lingual region of mandibular first molars between the cemento-enamel junction and alveolar bone crest) of Groups Placebo and Intervention, with comparisons between groups. * = Significant difference (Test t, *p*<0.05) when compared with Group Placebo. (B,C) Representative images of the specimens of the animals, stained with methylene blue in order to evidence the cemento-enamel junction and the bone crest. (B) Group Placebo; (C) Group Intervention.
(TIFF)
