# Supplementary figures and images for: Effects of the probiotic Bifidobacterium animalis subsp. lactis on the non-surgical treatment of periodontitis. A histomorphometric, microtomographic and immunohistochemical study in rats
Source: PLoS One. 2017 Jun 29;12(6):e0179946. doi: 10.1371/journal.pone.0179946 (PMC5491108; doi:10.1371/journal.pone.0179946)

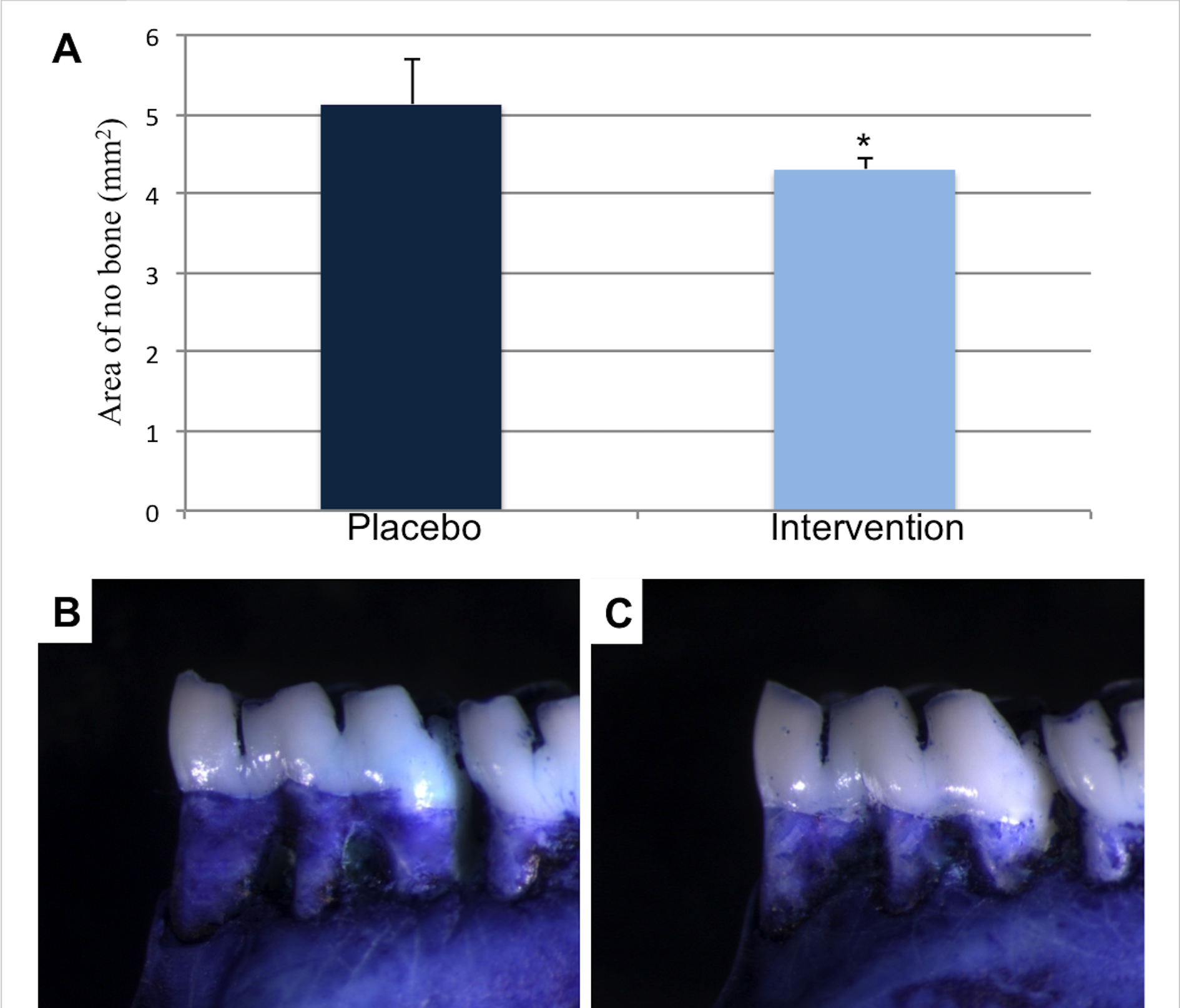

Supplement: S1 Fig — (A) Means and standard deviations of the Area (mm2) of No Bone (delimited in the lingual region of mandibular first molars between the cemento-enamel junction and alveolar bone crest) of Groups Placebo and Intervention, with comparisons between groups. * = Significant difference (Test t, p<0.05) when compared with Group Placebo. (B,C) Representative images of the specimens of the animals, stained with methylene blue in order to evidence the cemento-enamel junction and the bone crest. (B) Group Placebo; (C) Group Intervention. (TIFF) [file pone.0179946.s001.tiff]
